# Metabolic dysfunction-associated fatty liver disease exacerbates hematoma expansion in intracerebral hemorrhage: an explainable machine learning approach

**DOI:** 10.3389/fendo.2026.1912965

**Published:** 2026-08-26

**Authors:** Zhuo Yang, Jie He, Yong Song, Qiang Meng

**Affiliations:** 1 Department of Encephalopathy, Chengdu Xinjin District Hospital of Traditional Chinese Medicine, Chengdu, China; 2 Department of Comprehensive Neurology, Chengdu Xinjin District People’s Hospital, Chengdu, China; 3 Intensive Care Unit, Chengdu Xinjin District People’s Hospital, Chengdu, China

**Keywords:** hematoma expansion, intracerebral hemorrhage, machine learning, MAFLD, SHAP

## Abstract

**Background:**

Although hematoma expansion (HE) severely deteriorates outcomes in hypertensive intracerebral hemorrhage (HICH), predicting HE in patients comorbid with metabolic dysfunction-associated fatty liver disease (MAFLD) lacks specific machine learning tools. This study aimed to bridge this gap by exploring the interactive pathophysiological associations.

**Methods:**

Data from 529 HICH-MAFLD patients were used to construct five predictive algorithms. The best-performing model was subjected to SHapley Additive exPlanations (SHAP) to map variable interactions.

**Results:**

The XGBoost model achieved the best discrimination (AUC = 0.911). SHAP analysis revealed non-linear, synergistic statistical associations between the “liver-brain axis” components. Specifically, severe hepatic fibrosis (FIB-4 index) and systemic inflammation (hs-CRP) appeared to exponentially amplify the HE risk associated with local hemodynamic parameters (baseline hematoma volume and systolic blood pressure).

**Conclusion:**

The SHAP-integrated XGBoost algorithm suggests a robust predictive framework for early HE. The statistical synergy unmasked between metabolic inflammation and local hemodynamics highlights the potential clinical significance of the “liver-brain axis” in guiding individualized risk stratification.

## 1 Introduction

Intracerebral hemorrhage (ICH) is a devastating cerebrovascular event in the hypertensive population, and is linked to high morbidity and mortality (1). Early hematoma expansion (HE) is a major factor of secondary neurological deterioration and poor clinical outcome (2). It is therefore important to recognize those patients who are at high risk for HE in the acute phase (3). Although local hemodynamic fluctuations and intrinsic properties of the hematoma are known to play a role in the development of HE, it is now recognized as a more complicated process and possibly influenced by systemic physiological factors (4).

Metabolic dysfunction-associated fatty liver disease (MAFLD) is highly prevalent and shares the common features of chronic systemic inflammation, insulin resistance and endothelial dysfunction (5). Recent multi-omics and pharmacological studies have extensively highlighted that lipid metabolic disorders and subsequent hepatic fibrogenesis actively drive systemic inflammatory cascades (6–8). Liver fibrosis is reflected in subclinical changes in the coagulation system and in dysfunctional hemostasis in the advanced stages of MAFLD (9). These changes in the systemic pathophysiology may have theoretical implications on the vulnerability of the microvasculature of the brain after an acute rupture (10). The possible role of MAFLD in the evolution of HE in HICH patients, however, has been understudied in current cerebrovascular research.

The relationship between systemic metabolic inflammation and local cerebrovascular hemodynamics will most likely be non-linear and synergistic (11). The traditional predictive methods consisting mainly of linear regression analysis may not have the ability to adequately detect these high dimensional and complex interactive patterns (12). An alternative method for modelling these complex relationships is through the use of advanced machine learning algorithms (13). In recent years, clinical decision-making has increasingly evolved from traditional hypothetico-deductive models to knowledge-enhanced machine learning frameworks, which excel at integrating high-dimensional clinical and metabolic profiles (14). Tree-based ensemble algorithms, in particular, excel at deciphering hierarchical interactions and threshold effects without stringent assumptions about data distribution. However, these algorithms are often opaque and not readily interpretable, which makes it hard for doctors to apply the algorithm to an individual patient in an emergency situation.

The present study was undertaken to develop and test various machine learning models to predict early HE in a selected group of HICH patients with MAFLD. Moreover, the SHapley Additive exPlanations (SHAP) method was incorporated to obtain global and local interpretability. The aim of this research was to provide a better understanding of the comorbid pathophysiology and to provide a potentially explainable framework for individualized risk stratification by examining the possible non-linear synergies between liver metabolic markers and neurosurgical parameters. It was hypothesized that MAFLD may exacerbate hematoma expansion primarily through the synergistic pathways of systemic inflammation and underlying coagulation disorders.

## 2 Materials and methods

### 2.1 Study design and ethical approval

This single-center, retrospective observational study was approved by the Institutional Review Board of Chengdu Xinjin District Hospital of Traditional Chinese Medicine (Approval No.: KYSRKS202605) and analyzed clinical data collected at the hospital from January 2020 to December 2025. The ethical principles laid down in the 1975 Declaration of Helsinki were strictly followed in all research procedures employed in this study. The ethics committee formally waived the requirement for written informed consent because the study was retrospective and only anonymized patient information was analyzed.

### 2.2 Patient selection

Patients with hypertensive intracerebral hemorrhage (HICH) and metabolic dysfunction-associated fatty liver disease (MAFLD) were screened. The inclusion criteria were: (1) age ≥ 18 years, (2) spontaneous ICH on initial NCCT obtained within 24 hours of the onset of symptoms, (3) the presence of MAFLD that was diagnosed by international consensus criteria, and (4) a follow-up NCCT obtained within 72 hours of admission. Patients who had secondary ICH due to trauma, aneurysms, arteriovenous malformations, or brain tumors, had undergone surgery before the follow-up NCCT, or had severe primary hepatic or renal dysfunction (including non-MAFLD cirrhosis and active viral hepatitis), or who had significantly incomplete medical records or imaging data were excluded. Hematoma expansion (HE) was defined as the primary endpoint as per the current standard definition used in clinical trials: increase in absolute hematoma volume >6 mL, or >33% relative change between baseline and follow-up CT scan.

### 2.3 Data collection and clinical variables

Conceptualization of the clinical data into three analytical domains guided the abstraction of 15 distinct clinical variables from electronic medical records. Demographic factors and medical history were assessed in the first domain, including age, body mass index (BMI), and history of antiplatelet drug use. The second domain captured neurosurgical and hemodynamic parameters, including admission Glasgow Coma Scale (GCS) scores, admission systolic blood pressure (SBP), time from symptom onset to initial CT, CTA spot sign status, and baseline hematoma volume (calculated via the ABC/2 formula). A third domain was created to assess the metabolic, inflammatory, and hepatic profile using the following parameters: FIB-4 index, high-sensitivity C-reactive protein (hs-CRP), fasting blood glucose (FBG), triglycerides (TG), alanine aminotransferase (ALT), platelet count (PLT), and fibrinogen (FIB).

### 2.4 Machine learning algorithms and model construction

The data were first divided into a 70% training set and a 30% validation set with the aim to validate the feature predictiveness without changing the target class distribution. A 10-fold cross-validated Least Absolute Shrinkage and Selection Operator (LASSO) regression was first performed to select and reduce the dimensions of the variables, based on the tuning parameter (lambda) that yielded the minimum binomial deviance. Five predictive models were subsequently created for comparison after the critical features were identified. These modeled algorithms included Logistic Regression (LR), Decision Tree (DT), Support Vector Machine (SVM) with an RBF kernel, Random Forest (RF) with 500 trees, and eXtreme Gradient Boosting (XGBoost).The parameters of the XGBoost model were carefully selected through iterative search mechanisms to ensure the model learns efficiently and avoids overfitting. In particular, it had a maximum depth of 3, learning rate (eta) of 0.05, and 100 boosting rounds. Binary logistic regression was used for the objective function. During the training stage, class weight parameters (e.g. scale_pos_weight in XGBoost) were adjusted to make sure that the algorithm assigned appropriate weights to misclassifications of the minority class. After model finalization, the SHapley Additive exPlanations (SHAP) framework was applied in order to obtain the marginal contribution of each feature, and so to obtain both a global and a local interpretability for the predictions of the non-linear model.

### 2.5 Statistical analysis

All statistical evaluations were performed in the R programming environment. The Shapiro-Wilk test was first used to screen continuous variable distributions. This enabled us to report normally distributed data as means ± standard deviations (SD) and to use independent-samples t-tests, and to express skewed data as medians [interquartile ranges (IQR)] and apply Mann-Whitney U testing. Frequencies and percentages were used to summarize categorical features, and these were analyzed using Pearson’s Chi-square or Fisher’s exact test as appropriate for the data. Model discrimination and predictive strength were evaluated using the Area Under the Receiver Operating Characteristic Curve (AUC), Precision-Recall AUC (PR-AUC), accuracy, sensitivity, specificity, and F1-score. In addition, the DeLong test was used to evaluate any statistical differences in the AUC values between models. Statistical significance was established at a two-sided P-value < 0.05.

## 3 Results

### 3.1 Baseline characteristics and feature engineering

This study comprised 529 HICH patients comorbid with MAFLD, of whom 254 had hematoma expansion (HE) and 275 did not. The observed HE incidence (around 48.0%) was comparatively high compared to the prevalence of HE generally in the population. This might be due to the fact that MAFLD often occurs in the context of a systemic chronic inflammatory state and endothelial dysfunction with or without coagulopathy, and this comorbid population is at an ongoing risk of secondary bleeding.

Baseline characteristics by HE status are shown in **Table 1**. Univariate analysis showed that established neurosurgical and hemodynamic parameters differed significantly between the two groups: admission GCS score, admission SBP, baseline hematoma volume, time from onset to CT and the presence of CTA spot sign (all P < 0.05). Also, liver fibrosis marker FIB-4 was significantly higher in HE group. However, the univariate analysis did not show any statistically significant relationship between several metabolic and inflammatory markers (e.g., hs-CRP, fasting blood glucose, triglycerides, and platelet count) and hematoma expansion (P > 0.05). This indicates that the effect of these systemic metabolic factors on hematoma expansion may be complex non-linear synergistic effects with neurosurgical factors, as opposed to simple independent linear effects. The results highlight the need for using more sophisticated machine learning techniques to obtain higher-dimension relationships in data.

**Table 1:** Baseline characteristics of the study cohort (HICH patients comorbid with MAFLD), stratified by hematoma expansion.

Clinical variables	Total cohort (n=529)	Non-HE group (n=275)	HE group (n=254)	*P*-value
Demographics & medical history				
Age (years) (Mean ± SD)	61.6 ± 10.6	60.9 ± 10.5	62.3 ± 10.7	0.125
Body Mass Index (kg/m ²) (Mean ± SD)	27.8 ± 3.2	27.6 ± 3.2	28.1 ± 3.2	0.054
Antiplatelet Therapy History, n (%)	105 (19.8%)	53 (19.3%)	52 (20.5%)	0.813
Neurosurgical & hemodynamic parameters				
Admission GCS Score (Median[IQR])	11.0 [9.0-13.0]	12.0 [9.0-13.0]	11.0 [8.2-13.0]	0.022
Admission Systolic BP (mmHg) (Mean ± SD)	167.4 ± 21.6	163.1 ± 20.4	172.1 ± 22.0	<0.001
Baseline Hematoma Volume (mL) (Median[IQR])	22.3 [15.7-33.0]	17.1 [12.6-22.9]	30.8 [22.6-40.3]	<0.001
Time from Onset to CT (Hours) (Median[IQR])	3.0 [2.1-4.5]	3.5 [2.4-5.1]	2.7 [1.8-3.9]	<0.001
Spot Sign on CTA, n (%)	94 (17.8%)	32 (11.6%)	62 (24.4%)	<0.001
Metabolic, inflammatory & hepatic markers				
FIB-4 Index (Liver Fibrosis) (Median[IQR])	3.4 [2.4-4.5]	3.1 [2.1-4.4]	3.5 [2.7-4.7]	0.008
High-Sensitivity CRP (mg/L) (Median[IQR])	8.9 [5.6-12.5]	8.5 [5.3-11.9]	9.1 [5.9-12.9]	0.153
Fasting Blood Glucose (mmol/L) (Mean ± SD)	7.1 ± 1.8	7.2 ± 1.9	7.0 ± 1.7	0.259
Triglycerides (mmol/L) (Median[IQR])	2.1 [1.6-2.7]	2.1 [1.6-2.8]	2.0 [1.5-2.7]	0.400
Alanine Aminotransferase (U/L) (Median[IQR])	45.0 [32.0-65.0]	44.0 [31.0-62.5]	47.0 [33.0-66.8]	0.154
Platelet Count (×10^9/L) (Mean ± SD)	199.6 ± 54.1	196.5 ± 54.8	203.0 ± 53.2	0.166
Fibrinogen (g/L) (Mean ± SD)	3.5 ± 0.8	3.5 ± 0.8	3.5 ± 0.8	0.794

**Figure 1:**
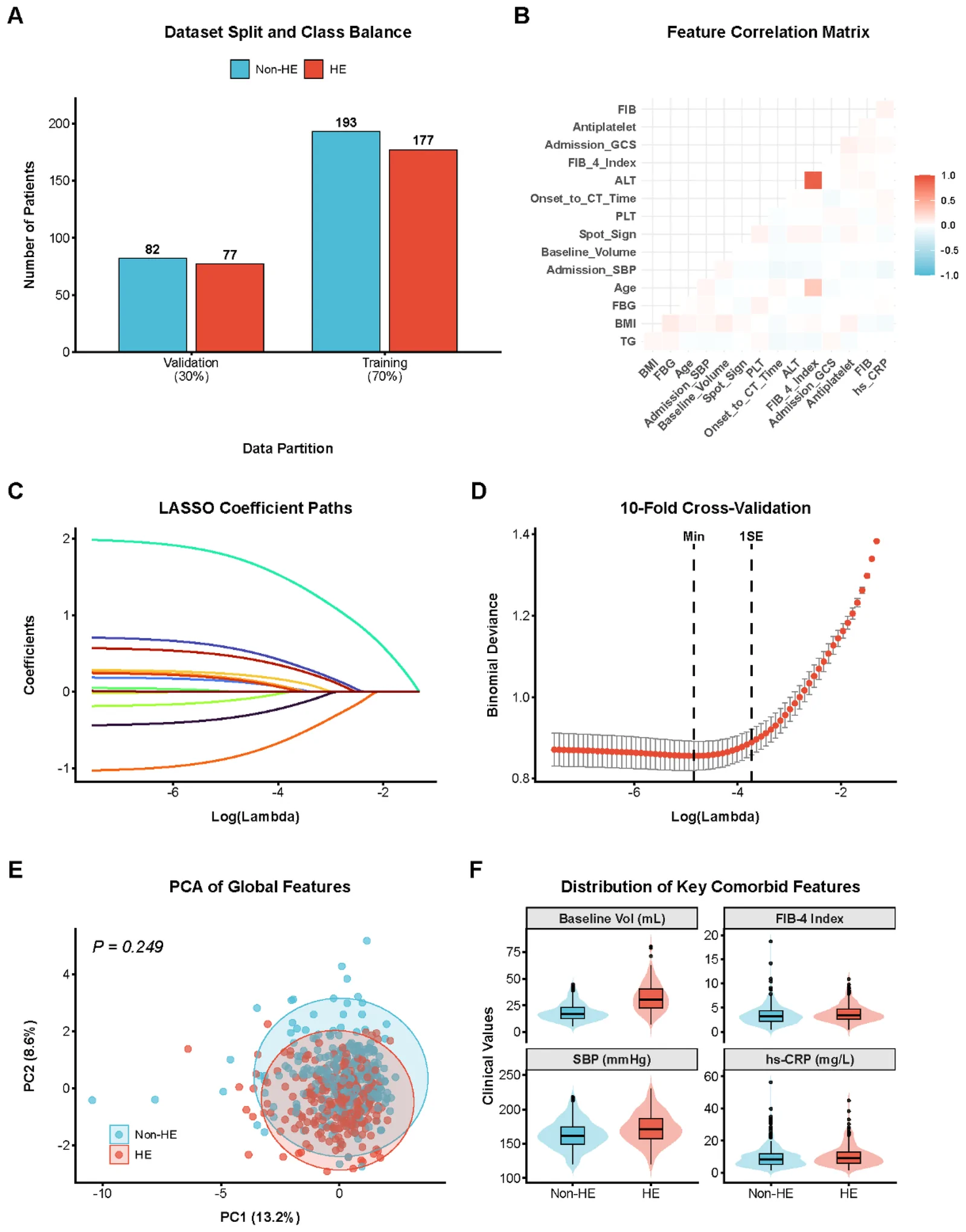
Dataset construction and feature selection. **(A)** Bar chart showing the distribution of hematoma expansion (HE) and non-HE cases in the training and validation cohorts. **(B)** Heatmap showing the correlation matrix among the clinical features. **(C)** Least Absolute Shrinkage and Selection Operator (LASSO) coefficient paths for feature selection. **(D)** Ten-fold cross-validation curve for the LASSO regression, indicating the optimal Log(Lambda) value. **(E)** Principal Component Analysis (PCA) scatter plot of the global features for HE and non-HE groups. **(F)** Violin and box plots illustrating the distributions of Baseline Volume, FIB-4 Index, Admission SBP, and hs-CRP between the two groups.

A training cohort (70%) and validation cohort (30%) was created for model development. The distribution of HE and non-HE classes was kept balanced between the two sets, as shown in **Figure 1A** to reduce possible sampling bias in the models training. Most of the clinical parameters were independent and there was no serious multicollinearity in the feature correlation analysis (**Figure 1B**). Significant physiological associations were seen mostly between FIB-4 index, age and ALT levels, which correspond with the formula used to derive the FIB-4 index.

To assess the shrinkage of feature coefficients and to determine the best combination of penalty parameters (**Figures 1C, D**), least absolute shrinkage and selection operator (LASSO) regression and a 10-fold cross-validation were used. Principal Component Analysis (PCA) was applied to evaluate the linear separability of the global clinical features. In the PCA scatter plot, as shown in **Figure 1E**, there was no appreciable separation between the HE and non-HE groups along the first principal component (P = 0.249), and there was a large overlap. This substantial overlap suggests a strong justification for using non-linear, tree-based machine learning algorithms to distinguish clinical outcomes in this comorbid population in future analysis. In addition, there was a visual comparison of the distribution of key neurosurgical and metabolic features (**Figure 1F**). Visualization showed that the distribution tails of the FIB-4 index and hs-CRP were longer in the HE group, suggesting a possible role of the metabolic inflammation risk-amplifier, as well as higher baseline volume and admission SBP.

### 3.2 Model development and performance evaluation

Based on the non-linear clinical associations and optimal features found in the previous analysis, we then proceeded to develop and thoroughly assess five different machine learning algorithms in the validation cohort. In particular, these models included Logistic Regression (LR), Decision Tree (DT), Support Vector Machine (SVM), Random Forest (RF), and eXtreme Gradient Boosting (XGBoost).

**Figure 2:**
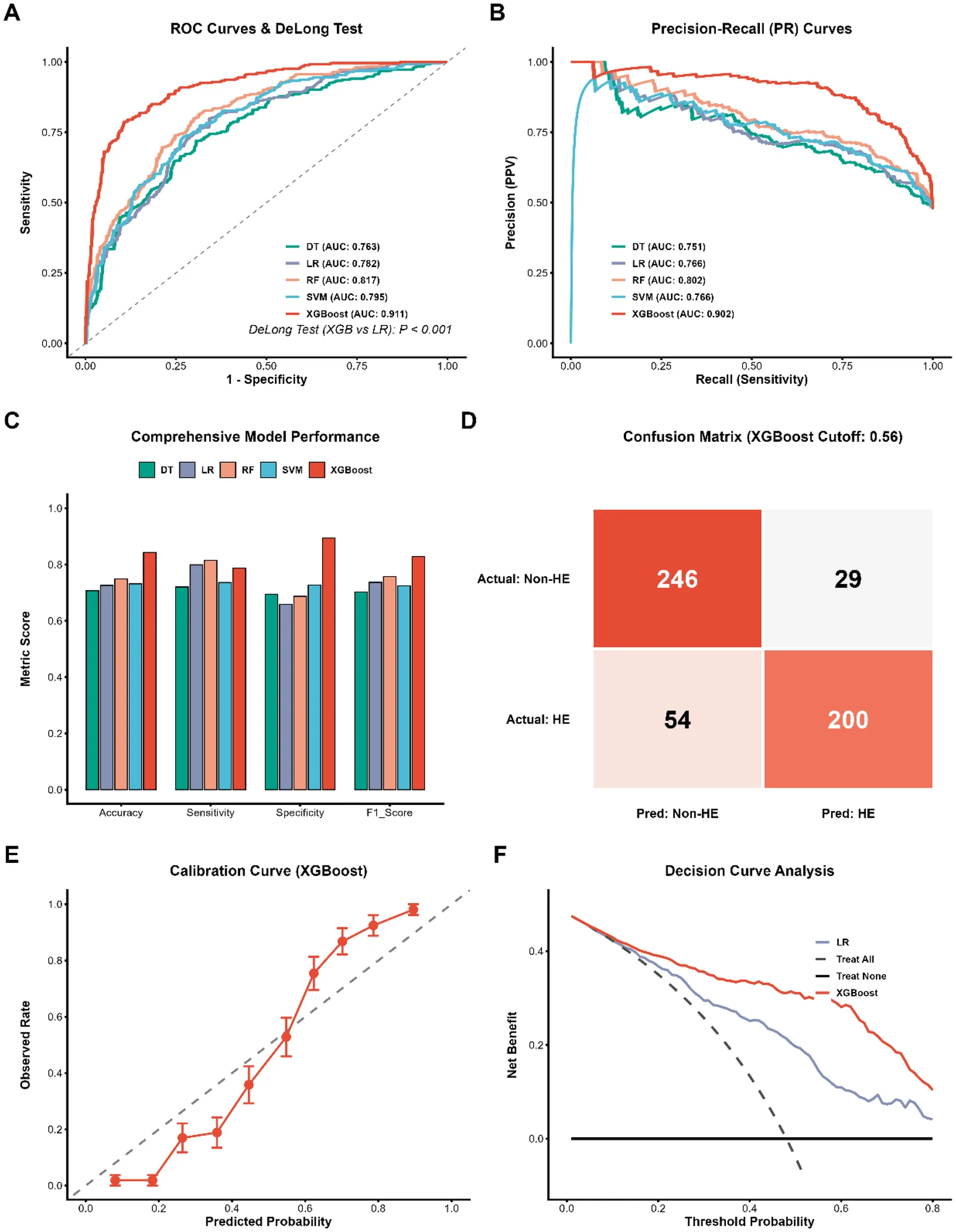
Performance evaluation of the machine learning models. **(A)** Receiver Operating Characteristic (ROC) curves of the five predictive models, with the DeLong test comparing XGBoost and Logistic Regression (LR). **(B)** Precision-Recall (PR) curves for the evaluated models. **(C)** Bar charts illustrating the accuracy, sensitivity, specificity, and F1-score of each model. **(D)** Confusion matrix for the XGBoost model at the optimal cutoff value. **(E)** Calibration curve suggesting the agreement between the XGBoost-predicted probability and the observed rate. **(F)** Decision Curve Analysis (DCA) showing the net clinical benefit of the models across different threshold probabilities.

The discriminative ability of each model was then graphically illustrated and numerically evaluated using Receiver Operating Characteristic (ROC) curves. As shown in **Figure 2A**, XGBoost model had the best AUC value of 0.911, whereas the traditional LR model had the lowest value of 0.782. The DeLong test showed that the difference in these two AUCs between the XGBoost and LR was statistically significant (P < 0.001). Precision-Recall (PR) curves were also plotted as an additional measure of precision for this model (where the class distribution was relatively balanced, **Figure 2B**). The XGBoost model continued to demonstrate predictive performance with a PR-AUC of 0.902, indicating a strong ability to correctly classify the true HE events while reducing the rate of false-positive results.

The comparison of the predictive metrics for the five models is given in a comprehensive manner in **Figure 2C**. The XGBoost algorithm had a favorable balance with all other algorithms, consistently high overall accuracy, sensitivity, specificity and F1 score. The confusion matrix for the XGBoost model was created with an optimized probability threshold of 0.56 (derived from the Youden Index) to give a detailed idea of the classification performance (**Figure 2D**). The threshold value resulted in 78.7% sensitivity and 89.4% specificity in correctly identifying 200 true HE cases and 246 non-HE cases. The number of false positives (n = 29; low predicted probability, high actual probability) is relatively small indicating that the model is not likely to result in over-treatment, and the number of false negatives (n = 54; low predicted probability, low actual probability) may represent a limit to the present feature set and inherent complexity of HE pathogenesis.

In addition to discrimination, the reliability of the predicted probabilities was also evaluated by using a calibration curve (**Figure 2E**). The plot showed that the XGBoost-predicted probabilities were consistent with the observed HE frequencies for different risk deciles with low probability drift and hence could be interpreted in a clinical setting. Finally, Decision Curve Analysis (DCA) was used to assess the potential clinical usefulness of the models. The XGBoost model demonstrated a higher net clinical benefit compared to the traditional LR model and baseline scenarios (all or none treatment) over a wide range of threshold probabilities (from 0 to 0.8) as shown in **Figure 2F**. This discovery indicates that the XGBoost model could be a practical solution for improving early intervention in high-risk comorbid patients in the clinical pathway.

### 3.3 Global interpretability of the XGBoost model via SHAP analysis

After confirming that our XGBoost model had predictive superiority, we then proceeded to transparently assess how each of the clinical parameters globally influenced the algorithmic output using the SHapley Additive exPlanations (SHAP) approach. Although this technique is highly effective in highlighting the importance of features, it is important to bear in mind that SHAP metrics represent only statistical associations and cannot be interpreted as direct biological causes.

**Figure 3:**
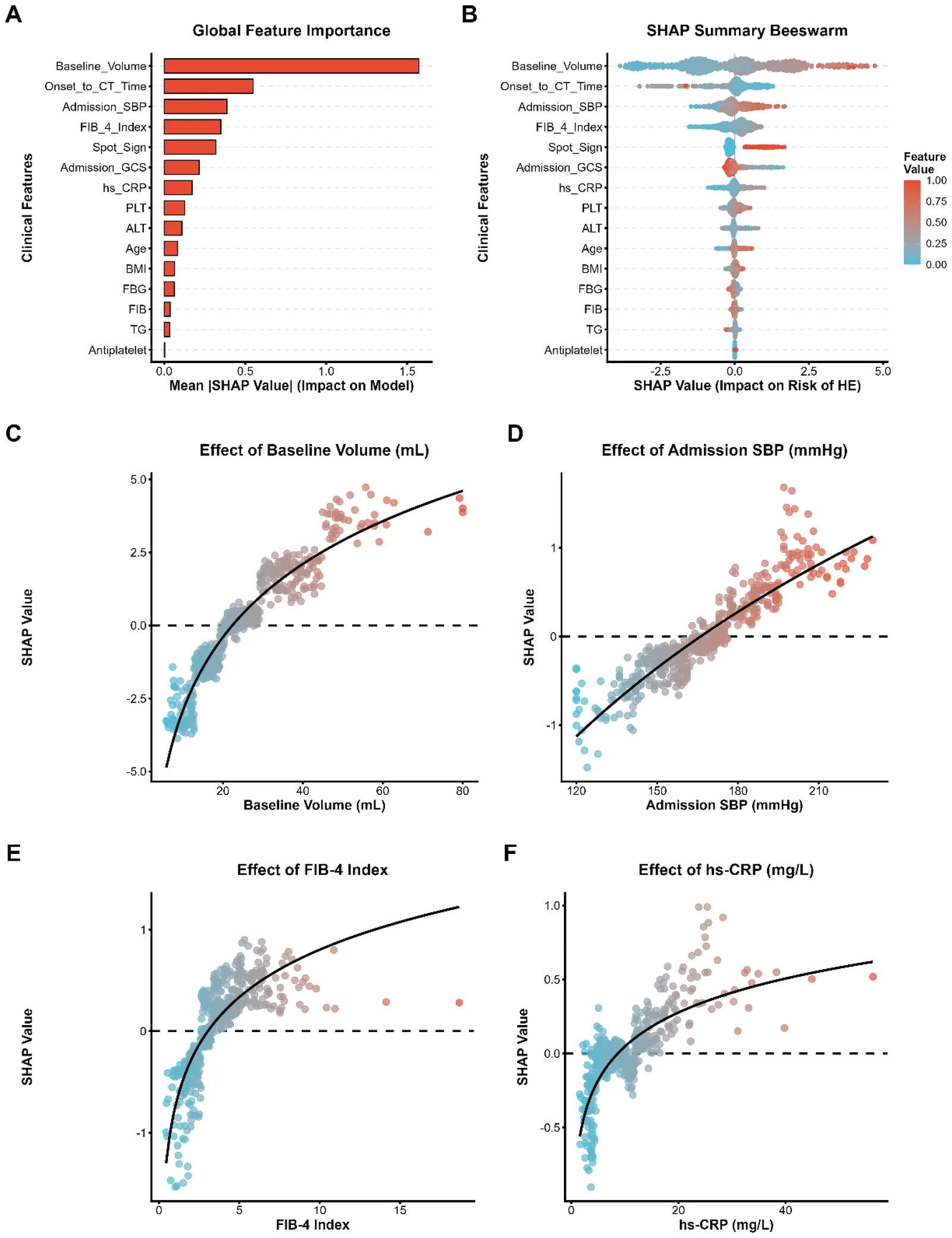
Global interpretability of the XGBoost model using SHAP analysis. **(A)** Global feature importance ranked by mean absolute SHAP values. **(B)** SHAP summary bee swarm plot indicating the directional impact of feature values on the predicted risk of HE. **(C–F)** SHAP dependence plots illustrating the individual non-linear effects of Baseline Hematoma Volume **(C)**, Admission SBP **(D)**, FIB-4 Index **(E)**, and hs-CRP **(F)** on the model output.

The importance of features is ranked using the average absolute SHAP values on the global scale (**Figure 3A**). The FIB-4 index was the fourth most important contributor, with classical neurosurgical parameters (baseline hematoma volume, time from onset to CT and admission SBP) predictably emerging as the top three. This fairly high ranking indicates that liver fibrosis could be a significant co-contributory risk factor in this particular comorbid population.

The directional effect of these features is further shown using the SHAP summary bee swarm plot (**Figure 3B**). Baseline volume, admission SBP, and FIB-4 index had all high SHAP values and were positively associated with predicted probability of HE (red dots). In contrast, longer time from onset to CT (red dots) were associated with negative SHAP values, which resulted in a prediction of reduced risk. This discovery is consistent with the clinical observation that hematomas are likely to settle down and stabilize with time and that late-presenting patients are unlikely to develop subsequent enlargement.

SHAP dependence plots were created to depict the complex non-linear relationships. The trend lines were fitted using logarithmic regression, a choice whose goal is to model possible threshold and saturation effects and reduce artefacts of the extrapolation at the tails of the data distributions. As shown in **Figure 3C**, the SHAP value for baseline volume was steeply increasing up to ~40mL and then leveling off. Admission SBP showed almost linear, positive and consistent relationships with the predicted risk (**Figure 3D**).

In addition, the dependence plots of the FIB-4 index (**Figure 3E**) and hs-CRP (**Figure 3F**) showed different threshold patterns. The corresponding risk increased quickly at lower levels of these metabolic/inflammatory measures, and then leveled off at higher levels. This plateau pattern indicates that the effect on the predicted risk of hematoma expansion may be “maxed out” and constant for hepatic impairment or systemic inflammation once a certain level of severity is reached.

### 3.4 Deep dive into comorbidity interactions and clinical phenotyping

**Figure 4:**
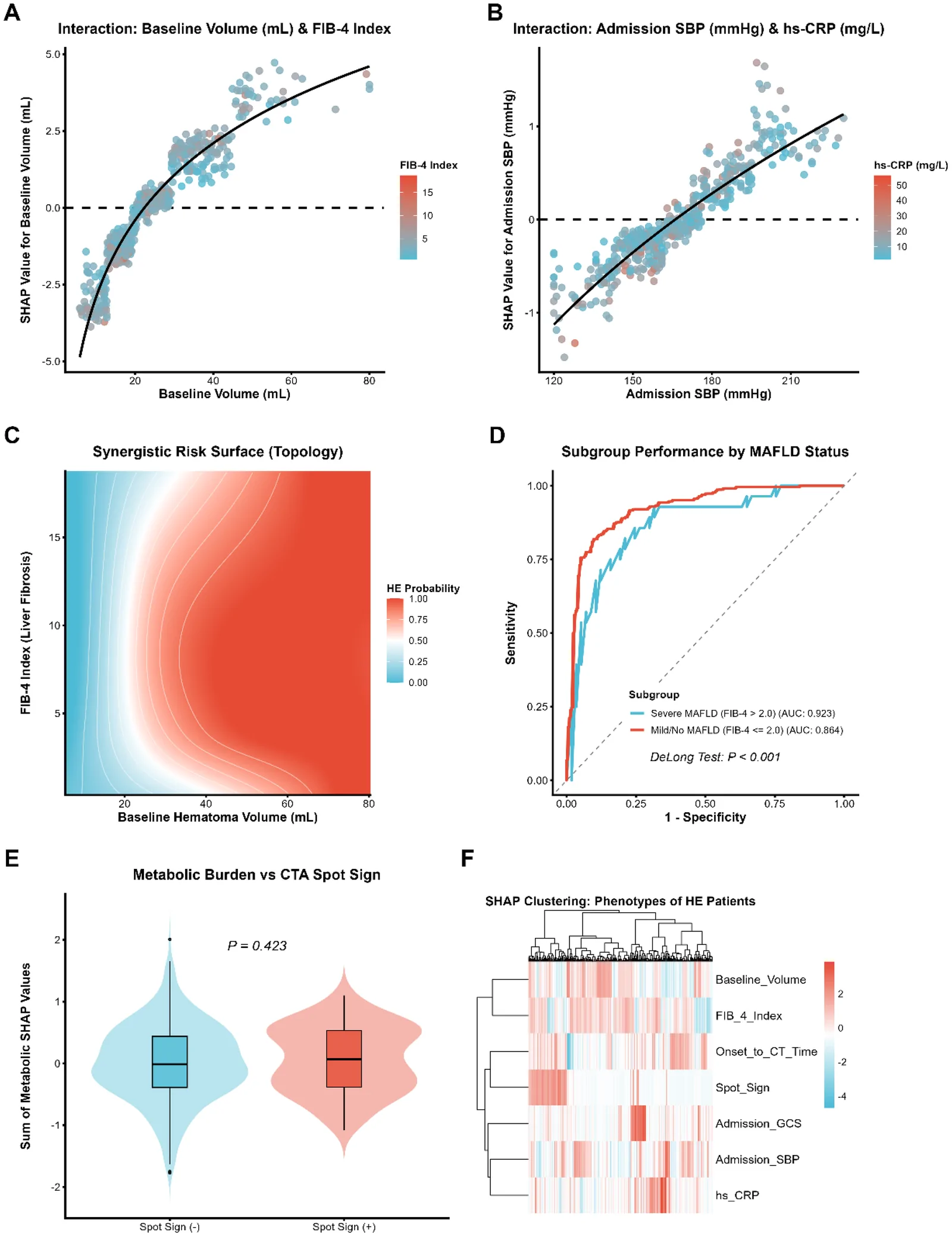
Feature interaction and subgroup analysis. **(A, B)** SHAP interaction dependence plots suggesting the interplay between Baseline Volume and FIB-4 Index **(A)**, and Admission SBP and hs-CRP **(B)**. **(C)** A 2D synergistic risk surface contour plot demonstrating the combined effect of Baseline Volume and FIB-4 Index on the predicted HE probability. **(D)** ROC curves indicating the predictive performance of the model stratified by MAFLD severity subgroups. **(E)** Violin plot comparing the sum of metabolic SHAP values between patients with and without the CTA spot sign. **(F)** Hierarchical clustering heatmap of SHAP values revealing potential clinical phenotypes of patients with HE.

Building upon the global feature importance, we have also explored possible non-linear synergistic effects between the clinical variables. The SHAP interaction dependence between baseline hematoma volume and FIB-4 is shown in **Figure 4A**. Within a similar baseline volume range (e.g., 30–40 mL), patients with higher FIB-4 index (represented by red dots) had higher SHAP values, compared to those with lower FIB-4 index. Similar interaction was seen between admission SBP and hs-CRP (**Figure 4B**) such that, the combination of high blood pressure and systemic inflammation was linked to an additional higher shift in the SHAP value assigned.

A two-dimensional synergistic risk surface was created from the predicted probabilities, to macroscopically observe the combined effect of the liver-brain axis components (**Figure 4C**). The topological contour shows a gradient from low-risk area (blue zone, low volume, low FIB-4) towards the high-risk area (dark red zone). Based on the density of the contour lines, the predicted probability of hematoma expansion appears to increase in a non-linear manner when both neurological and hepatic deficits are simultaneously poor.

To assess the ability of the model to capture these metabolic interactions in clinical situations, a subgroup ROC analysis, based on the severity of MAFLD was performed (**Figure 4D**). The model was more discriminative in the severe MAFLD group (FIB-4 > 2.0; AUC: 0.923) than in the mild/no MAFLD group (AUC: 0.864) with a significant DeLong test (P < 0.001). This performance difference implies that the inclusion of the complex metabolic-inflammatory profiles is advantageous for the machine learning algorithm and especially useful for assessing high-risk patients with complicated comorbidities.

We also evaluated the correlation of the sum of the metabolic SHAP values with the CTA spot sign (**Figure 4E**). Interestingly, there was no significant difference between two groups (P = 0.423). This is not a model limitation but rather suggests that the systemic metabolic burden is likely independent from the local microvascular rupture (spot sign) and is a dimension of risk. The strong predictive power of the model might be due to the fact that it can model both non-overlapping pathways at the same time.

Finally, an unsupervised hierarchical clustering heatmap was created from the SHAP profiles of the patients who did actually have hematoma expansion (**Figure 4F**). The dendrogram suggests that there are potential clinical phenotypes. Some patient clusters showed a risk profile that was mainly led by hemodynamic factors (prominent red block in baseline volume and SBP), while other clusters had a risk profile that was mainly led by metabolic and inflammatory factors (prominent red block in FIB-4 and hs-CRP). This phenotyping reveals the co-morbid population’s underlying heterogeneity of hematoma expansion.

### 3.5 Local interpretability for individualized risk assessment

**Figure 5:**
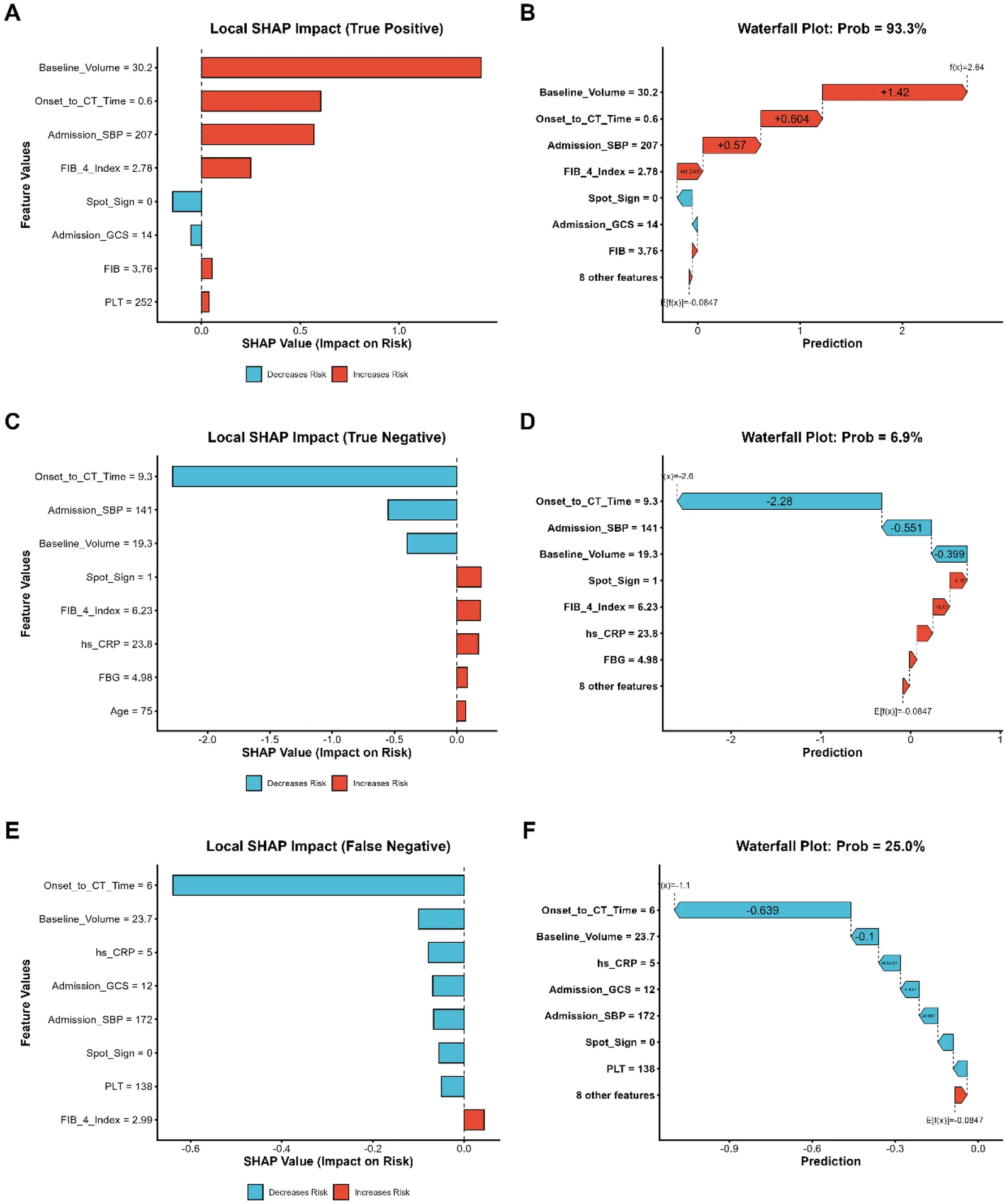
Local SHAP interpretability for individual patient predictions. Local SHAP impact bar plots (left column) and corresponding waterfall plots (right column) for three representative cases: **(A, B)** a true positive case, **(C, D)** a true negative case, and **(E, F)** a false negative case. Red bars/arrows indicate features that may push the prediction toward a higher risk, while blue bars/arrows indicate features pulling the prediction toward a lower risk relative to the baseline expectation.

To operationalize the findings at the population level for clinical use, local SHAP explanations were created, visualizing the model’s workflow of individual patient profiles. **Figure 5** shows the impact bar plot and waterfall plot for three representative cases locally. These waterfall plots show how certain feature values successively move the predicted probability away from the baseline expectation towards the final output (either push – red, or pull – blue).

True positive cases with high predicted probability of hematoma expansion (93.3%) are shown in **Figures 5A, B**. The high risk in this case was mainly attributed to a high baseline hematoma volume (30.2 mL), very short time from onset to CT (0.6 hours), high systolic BP on admission (207 mmHg), and high FIB-4 index (2.78). This profile corresponds with the synergistic high-risk phenotype that was being identified in the previous interaction analysis, with the combined hemodynamic-hepatic burdens leading to the occurrence of hematoma expansion.

On the other hand, **Figures 5C, D** illustrate a genuine negative in which the model was successful in labelling a low expansion probability (6.9%). The waterfall plot shows that although there were minor factors that increased the risk, there was good stabilization by other parameters such as significantly delayed presentation time (9.3 hours), well controlled admission SBP (141 mmHg) and moderate baseline volume (19.3 mL) which anchored the overall prediction very strongly towards safety. This indicates that the algorithm can provide correct weighting for strong clinical stabilizing factors and for baseline metabolic changes.

To assess the limitations of the model, a case of false negative was also explored (**Figures 5E, F**). In this instance the patient had a true hematoma expansion and the model predicted a relatively low probability (25.0%). The local SHAP analysis reveals that some apparently protective features, such as a delayed CT time (6 hours), a moderate hematoma volume (23.7 mL), and a low systemic inflammatory status (hs-CRP 5 mg/L) had a strong impact on the model’s decision. The FIB-4 index was also somewhat high, but mathematically such factors outweighed. This misclassification illustrates the heterogeneity of intracerebral hemorrhage. It suggests that the current feature set of hemodynamic and metabolic mechanisms may not fully explain hematoma expansion in a small number of outlier cases, but rather that other mechanisms may occur, either in the form of cerebral amyloid angiopathy or sporadic local vascular fragility.

## 4 Discussion

Hematoma expansion is a crucial component of clinical deterioration in intracerebral hemorrhage (ICH) under the background of hypertension, and the time course of hematoma expansion in ICH with metabolic dysfunction-associated fatty liver disease (MAFLD) is poorly characterized (15, 16). In the current study, an extreme gradient boosting model was created, which showed that the risk of expansion predicted by the model is non-linear with respect to systemic metabolic inflammation and that locally, systemic metabolic inflammation is synergistically increased by hepatic fibrosis, which also acts as a positive risk factor itself, and baseline hematoma volume.

Linear logistic regression is often used in conventional predictive models, and has proven useful in predicting other intracerebral hemorrhage complications such as early acute hydrocephalus (17). However, in our study, we found that this method might not fully capture the complexity of comorbid pathophysiological states (18). Compared to previously published predictive scores in general intracerebral hemorrhage populations, which typically achieve AUCs ranging from 0.60 to 0.88, our XGBoost model demonstrated highly competitive discriminative capacity (AUC: 0.911) (19). Interestingly, systemic markers like high sensitivity CRP and fasting blood glucose were not statistically significant in the univariate analysis, which could be considered an indication for methodological concern. Interestingly, the XGBoost algorithm showed better discriminative capacity, suggesting that the impact of these metabolic parameters on hematoma expansion is probably more complex and involves high dimension and non-linear synergistic pathways than a linear contribution. This is consistent with recent computational health studies that have shown that machine learning is especially beneficial in the analysis of complex clinical profiles that are difficult to explain with an additive model (20). The necessity of employing advanced computational models in this context is further supported by recent advancements in metabolic research. The application of machine learning, digital twin technology, bioinformatics analysis, and nomograms has increasingly demonstrated immense potential in the early diagnosis, personalized nutrition, and management of MAFLD (21–25). Furthermore, recent literature heavily emphasizes the critical role of the metabolic-inflammatory axis in MAFLD progression. Various novel glucose-lipid metabolism indicators (such as the TyG index and FAI), wrist circumference, and systemic inflammatory markers have been robustly associated with hepatic fibrosis and the severity of MAFLD (26–30). It is now widely recognized that the systemic burden of this metabolic dysfunction extends far beyond the liver, exacerbating subclinical damages in other organ systems, including left ventricular dysfunction, chronic kidney disease, and cerebrovascular vulnerability [Ref 11, Ref 12]. Consistent with these evolving concepts, our XGBoost model successfully captured the compounding effect of these multi-dimensional metabolic-inflammatory traits on the rapid deterioration of intracerebral hemorrhage.

**Figure 6:**
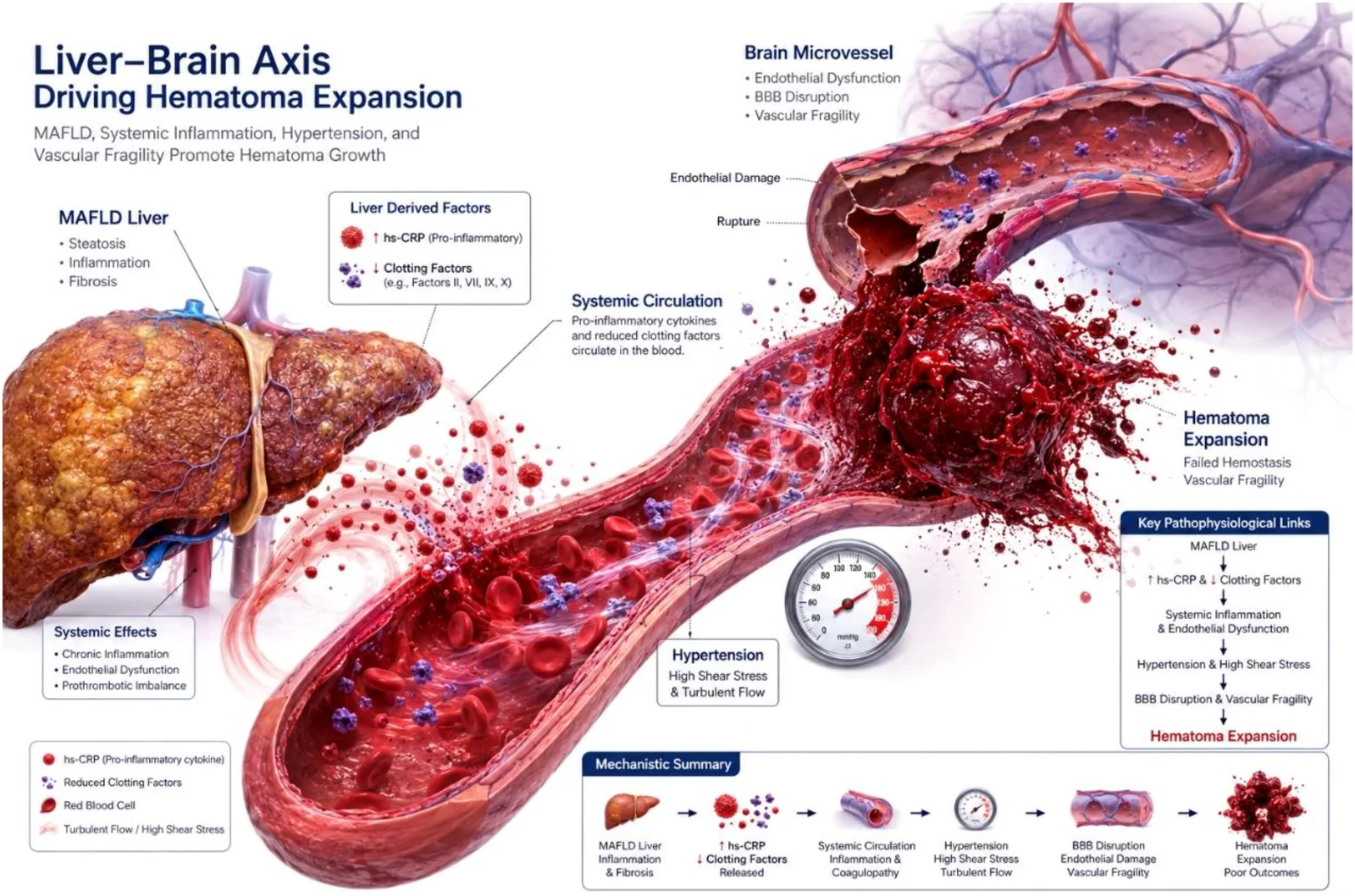
Proposed schematic representation of the “liver-brain axis” bridging MAFLD and hematoma expansion. This hypothetical pathway suggests that systemic inflammation (e.g., elevated hs-CRP) and impaired coagulation associated with hepatic fibrosis may synergistically compromise blood-brain barrier (BBB) integrity, thereby increasing microvascular vulnerability to local hemodynamic stressors (e.g., hypertension and high shear stress), ultimately leading to failed hemostasis and massive hematoma expansion.

The use of SHAP analysis aided in elucidating these complex relationships, which depicts statistical association but not necessarily biological causation (31). The dependence plots showed that there were threshold and saturation effects for the FIB-4 index and systemic inflammatory markers. The results of this study suggest that the effect of hepatic impairment or inflammation on the predicted risk of hematoma expansion may be more complex than the linear risk trajectory previously reported in other studies (32). Clinically, the rapid escalation of risk at lower levels prior to reaching the saturation plateau may indicate the lowest critical intervention threshold. This suggests that even mild elevations in these metabolic indices may warrant prompt and aggressive risk stratification and targeted management before the pathophysiological damage is maximized. Additionally, the topological interaction surface suggests a synergic association between liver fibrosis parameters, where moderate baseline hematoma risk seems to be exponentially increased in the context of high liver fibrosis parameters (33). Concurrently, the significant predictive weight of admission systolic blood pressure underscores the critical role of acute hypertension, which may act as a potent mechanical driver promoting the rupture of already vulnerable microvasculature. This pattern is compatible with a liver-brain axis, in which endothelial dysfunction and the presence of subclinical coagulopathy occurring with advanced fatty liver disease may contribute to increased vulnerability of the local microvasculature in the setting of acute hemodynamic changes (34, 35). Biologically, intense metabolic inflammation may lead to an excessive release of circulating cytokines that degrade the blood-brain barrier, while advanced hepatic fibrosis could impair the synthesis of essential coagulation factors. Together, these systemic deficits may synergistically and non-linearly compound the local mechanical stress exerted by the baseline hematoma, thus facilitating secondary bleeding (**Figure 6**).

The local SHAP explanations show how the model can be used in practice to get a view of the individual risk, whilst providing objective insight into the limits of the model. The detailed examination of this false-negative prediction demonstrates that there is a subset of hematoma expansions that occur despite seemingly protective metabolic and temporal profiles. Algorithmic misclassifications of this type imply that there is pathophysiological heterogeneity that is not measured by standard macroscopic and biochemical evaluations. This underlying heterogeneity is consistent with literature highlighting the intricate and multifactorial mechanisms driving non-vascular structural-related hemorrhages and complex ventricular extensions (36, 37), perhaps related to cerebral amyloid angiopathy or random vascular abnormalities. This further suggests that the absence of continuous coagulation monitoring markers and dynamic radiomics features may restrict the model’s capacity to predict nuanced clinical deterioration. But it is important to be aware of these limitations in model interpretability when thinking about how to responsibly operationalize predictive algorithms in the real world of clinical process.

This study is subject to several limitations. First, due to its retrospective single-center design and the lack of a non-MAFLD control group, potential sources of selection bias may exist, making it unclear whether the model’s performance is exclusively driven by MAFLD pathology. Second, the notably high hematoma expansion rate (48.0%) indicates a specialized high-risk cohort; thus, the model requires rigorous external validation in independent, multi-center cohorts with varied expansion rates to prove its generalizability prior to clinical application. Third, there are limited measurements of factors contributing to the coagulation cascade, an absence of comprehensive renal profile markers, and a lack of dynamic radiomics imaging features, and no dynamic neuroimaging parameters measured continuously, which may be able to give further mechanistic insight into what is causing the microvascular deterioration associated with hematoma progression.

## 5 Conclusion

In conclusion, based on the findings of this study, machine learning algorithms, especially XGBoost combined with SHAP analysis, could serve as a powerful tool to predict hematoma expansion in patients with HICH and MAFLD. The results suggest that systemic metabolic inflammation measures and hepatic fibrosis measures may act in a non-linear and synergic way with other local hemodynamic measures, enhancing the overall risk profile. In addition, explainable AI at the individual level could offer insights into the pathophysiological heterogeneity of this comorbid patient population, potentially helping physicians with early risk stratification in this population, albeit with caution. The current design is retrospective and single center and therefore the statistical associations do not necessarily imply biological causality. It is essential to emphasize that prospective external validation in independent, multi-center cohorts is a prerequisite before this model can be safely adopted in clinical applications. Future studies are needed in the future to externally validate these predictive patterns and to critically test the feasibility of their use in routine clinical decision making.

## Data Availability

The raw data supporting the conclusions of this article will be made available by the authors, without undue reservation.
